# A comparison of the molecular subtypes of triple-negative breast cancer among non-Asian and Taiwanese women

**DOI:** 10.1007/s10549-017-4195-7

**Published:** 2017-03-15

**Authors:** Ling-Ming Tseng, Jen-Hwey Chiu, Chun-Yu Liu, Yi-Fang Tsai, Yun-Lin Wang, Chu-Wen Yang, Yi-Ming Shyr

**Affiliations:** 1 Comprehensive Breast Health Center & Division of General Surgery, Department of Surgery, Taipei Veterans General Hospital, No. 201, Sec. II, Shih-pei Road, Taipei, 112 Taiwan, ROC; 2 Department of Surgery, School of Medicine, National Yang-Ming University, Taipei, Taiwan, ROC; 3 Institute of Traditional Medicine, School of Medicine, National Yang-Ming University, Taipei, Taiwan, ROC; 4 Division of General Surgery, Department of Surgery, Cheng-Hsin General Hospital, Taipei, Taiwan, ROC; 5 Division of Medical Oncology, Department of Oncology, Taipei Veterans General Hospital, Taipei, Taiwan, ROC; 6 School of Medicine, Institute of Clinical Medicine, National Yang-Ming University, Taipei, Taiwan, ROC; 7 Department of Microbiology, Soochow University, Taipei, Taiwan, ROC

**Keywords:** Triple negative, Breast cancer, Subtype, Gene expression, Precision medicine

## Abstract

**Background:**

“Precision medicine” is a concept that by utilizing modern molecular diagnostics, an effective therapy is accurately applied for each cancer patient to improve their survival rates. The treatment of triple-negative breast cancer (TNBC) remains a challenging issue. The aim of this study was to compare the molecular subtypes of triple-negative breast cancer (TNBC) between Taiwanese and Non-Asian women.

**Methods:**

GEO Datasets for non-Asian (12 groups, *n* = 1450) and Taiwanese (3 groups, *n* = 465) breast cancer, including 617 TNBC, were acquired, normalized and cluster analyzed. Then, using TNBC cell lines of different subtypes, namely, MDA-MB-468 (basal-like1, BL1), MDA-MB-231 (mesenchymal stem like, MSL), BT-549 (mesenchymal, M), MDA-MB-453 (luminal androgen receptor, LAR), and DU4475 (immunomodulatory, IM), real-time PCR in triplicate for 47 genes signatures were performed to validate the specificity of these subtypes.

**Results:**

The results showed that the percentage of TNBC subtypes in non-Asian women, namely, BL1, BL2, IM, M, MSL, and LAR was 13.56, 8.91, 16.80, 20.45, 8.30, and 11.13%, respectively. When data from Taiwanese were normalized and clustered, five TNBC subtypes, namely, BL (8.94%), IM (13.82%), M (22.76%), MSL (30.89%), and LAR (23.58%), were classified. Real-time PCR validated the specificity of these subtypes. Besides, the presence of interaction between IM- and MSL-subtypes suggests the involvement of tumor microenvironment in TNBC subtype classification.

**Conclusion:**

Our data suggested that there exist different presentations between non-Asian and Taiwanese TNBC subtypes, which provides important information when selection of therapeutic targets or designs for clinical trials for TNBC patients.

**Electronic supplementary material:**

The online version of this article (doi:10.1007/s10549-017-4195-7) contains supplementary material, which is available to authorized users.

## Introduction

Precision medicine has become an important emerging approach to the diagnosis, treatment, and prevention of disease, especially cancers; it takes into account the individual variability of each person in terms of genes, environment, and lifestyle. Breast cancer is the most common malignancy in women [1, 2]. Owing to tumor heterogeneity caused by cell phenotype diversity, different approaches to treatment and prognosis have been shown to be highly correlated with the intrinsic subtypes of the breast cancer [3]. Triple-negative breast cancer [TNBC, ER(−), PR(−), HER2(−)], which accounts for about 15% of breast cancers worldwide, is characterized by aggressive tumor behavior and a strong resistance to ant hormone treatment, chemotherapy, and targeted therapy [4–6].

Previously, using whole-genome (genome wide) analysis, including gene expression analysis (gene expression profiling), various TNBC molecular subtypes have been further identified. For example, six specific subtypes, namely, basallike1 (BL1), basallike2 (BL2), mesenchymal (M), mesenchymal stem like (MSL), immune response (M), and luminal androgen receptor positive (LAR) were first described by Lehmann et al. [7]. Since then, more investigations have targeted TNBC tumor heterogeneity using gene ontology [8–10], therapeutic targets [11, 12], and using mRNA or long noncoding RNAs (lncRNAs) as diagnostic criteria [13]. Although the six subtype classification has been refined recently [14, 15], the variation in molecular classification of TNBC across various different populations remains to be elucidated.

Accumulating evidence has shown that social economic, epidemiological, and genetic factors all play roles in tumor behavior, cancer subtype, and the prognosis of patients among different racial/ethnic groups [16–18]. For example, women of African heritage, compared to women of Caucasian heritage, have a higher rate of TNBC and a lower rate of receptor (+)/HER2(−) breast cancers after the age of 35 years [19]. Furthermore, a high prevalence and poorer clinical outcomes have been observed among African-American women with TNBC than among women of European descent [20, 21]. There is consensus that genome-wide studies, such as gene expression profile analysis, provide multi-gene signatures that are closely linked to TNBC carcinogenesis [22, 23]. Previous studies have demonstrated a significant association between the PTEN mutation, a high Ki67 index and the CD44^+^/CD24 phenotype among African-American women with TNBC [24]. In addition to the above findings, it has also been noted that there are frequently variations in the EGFR-activating mutations found in TNBCs among East Asians patients and this is not true for European patients [25]. In the context of these findings, controversy exists regarding the amount of variations that occurs in genomic profiles between different ethnic populations [26]. Therefore, the aim of the present study was to compare the molecular subtypes of triple-negative breast cancers (TNBCs) between Taiwanese female patients and nonunion female patients.

## Methods

### Subjects

Under the approval of the Institutional Review Board (# 201310020BC) of Taipei Veterans General Hospital, Taiwan, ROC, a total of 57 patients between June 2013 and September 2015 with TNBC [ER(−), PR(−), HER2(−)] were identified by immunohistochemical analysis of their pathological specimens. Total RNA was extracted from these TNBC tissue samples, and the RNA samples were used to conduct oligonucleotide microarray analysis by the Genome Research Center, National Yang-Ming University [27].

### Data set collection and TNBC identification by bimodal filtering

GE profiles from fourteen publicly available breast cancer microarray datasets, including twelve nonunion and two Taiwanese datasets (Sun Yat-Sen Cancer Center and Cathy hospital) (GEO, http://www.ncbi.nlm.nih.gov/gds; Array Express, http://www.ebi.ac.uk/microarrayas/ae/) were compiled and these were added to our dataset (GSE95700) (Supplementary Reference 1). In total, 1915 human breast cancer samples were included and among these samples a total of 617 TNBCs were identified (Table 1). The GE raw values for each of the datasets were normalized independently using the RMA procedure. The Affymetrix probes used for ER, PR, and HER2 were 205225_at, 208305_at, and 216836_s_at, respectively. A two-component Gaussian mixture distribution model was used to analyze the empirical expression distributions of ER, PR, and HER2 and the default parameters were estimated by maximum likelihood optimization using R statistical software (https://www.rproject.org/). After the posterior probability of a negative expression state for ER, PR, and HER2 had been estimated, a sample was defined as having negative expression if the posterior probability was less than 0.5. This process was followed by bimodal filtering to remove all ER/PR/HER2 positive tumors. The remaining TNBC tumors were then normalized along with positive controls for ER, PR, and HER2. Only samples that displayed a marked reduction in expression based on the above criteria compared to the positive controls were classified as TNBC (*n* = 617).

**Table 1:** Triple-negative breast cancer (TNBC) distribution in publicly available data sets

Non-Asian			Country	Taiwanese		
GEO accession	BC case	TNBC		GEO accession	BC case	TNBC
GSE12276	204	67	Netherlands	GSE20685	327	57
GSE14017	29	13	USA	GSE48390	81	16
GSE17907	51	1	France	GSE95700 (VGH)	57	50
GSE18864	84	53	Denmark			
GSE19615	115	35	USA			
GSE19697	24	24	USA			
GSE20711	88	24	Canada			
GSE21653	266	91	France			
GSE31448	353	131	France			
GSE42568	104	32	Ireland			
GSE43502	25	19	USA			
GSE58812	107	96	France			
Sum	1450	494		Sum	465	123

*BC* breast cancer, *TNBC* triple-negative breast cancer, *VGH* Veterans General Hospital

### Identification of TNBC subtypes

Previously, six distinct TNBC molecular subtypes were proposed by Lehmann et al. [7] and these were basallike1 (BL1), basallike2 (BL2), mesenchymal (M), mesenchymal stem-like (MSL), immune response (M), and luminal androgen receptor positive (LAR). Accordingly, using the published six type gene lists, we clustered and replotted the six types of heat map using our compiled complete dataset. In addition to background correction, the MAS5 procedure was applied to the Taiwanese data and then consensus clustering and *k*-means clustering were used to determine the optimal number of stable TNBC subtypes. Cluster robustness was assessed by consensus clustering using agglomerative k-means clustering using the average linkage for the 123 TNBC profiles based on the most differentially expressed genes (SD > 0.9; *n* = 5463 genes). The optimal number of clusters was determined from the Consensus Cumulative Distribution Function (CDF), which plotted the corresponding empirical cumulative distribution; this was defined over the range [0,1], and calculated based on the proportional increase in the area under the CDF curve. Following this, the number of clusters was decided when any further increase in cluster number (*k*) did not lead to a corresponding marked increase in the CDF area. Principal component analysis (PCA) and heat maps were generated using GeneSpring software (GeneSpring GX 11.5; Agilent Technologies, Inc., Santa Clara, CA, USA) and further pathway analysis was carried out using Ingenuity Pathway Analysis software [27] (IPA; Qiagen, Redwood City, CA, USA).

### Gene selection specific to each TNBC subtype

After consensus clustering and k-means clustering of the Taiwanese data, the TNBC subtypes were determined. The genes specific to each TNBC subtype were defined as followings: (1) the strongest probe with a fold change (ratio), >1.75 (upregulation) or <0.5 (downregulation), compared with the other subtypes; (2) the percentage of the sample with a GE difference >0 (sample GE − mean GE of other subtypes) of >80%; and a *p* value <10^4^ (*t* test: specific subtype versus other subtypes).

### Cell line and reagents

Under the approval of Institutional Review Board (# 201606012BC) of Taipei Veterans General Hospital, Taiwan, ROC, the human triple-negative breast cancer cell lines MDA-MB-468 (BL1), MDA-MB-231 (MSL), BT-549 (M), MDA-MB-453 (LAR), and DU4475 (IM) were obtained from the American Type Culture Collection (ATCC, Manassas, VA, USA), and these were then maintained in specific culture medium, namely F12 MEM (No. 12400024, Gibco, NY, USA), RPMI, as appropriate; the media were supplemented with 10% FBS, 2 mM l glutamine and penicillin/streptomycin, and the cells were cultured at 37 °C in a humidified atmosphere containing 5% CO_2_. Cells that were from three passages to ten passages were used.

### Total RNA extraction and reverse transcription PCR

Total RNA was isolated using the modified single step guanidinium thiocyanate method [28] (TRI REAGENT, T9424, Sigma Chem. Co., St. Louis, MO, USA). After the cells from the five different subtypes, namely, MDA-MB-468 (BL1), MDA-MB-231 (MSL), BT-549 (M), MDA-MB-453 (LAR), and DU4475 (IM) had been grown up and total RNAs extracted, complementary DNA (cDNA) was created using a First Strand cDNA Synthesis Kit (Invitrogen, CA, USA). TaqMan^®^ Gene Expression Assays were used to validate the differential expression at the mRNA level of the various identified genes sets that had been selected from consensus clustering results (Table 2). The TaqMan system was supported by a well-established primer database that reduces significantly the experimental failure due to inappropriate primer design.

**Table 2:** Gene list for validation of Taiwanese TNBC subtype

Subtype 1 (IM)		Subtype 2 (MSL)		Subtype 3 (M)		Subtype 4 (LAR)		Subtype 5 (BL)	
Probe	Gene symbol	Probe	Gene symbol	Probe	Gene symbol	Probe	Gene symbol	Probe	Gene symbol
232362_at^a^	CCDC18	227427_at	ARHGEF25	201268_at	NME1-NME2	218211_s_at	MLPH	219787_s_at	ECT2
206486_at	LAG3	206485_at	CD5	213801_x_at	RPSA	215465_s_at	ABCA12	231984_at	MTAP
207634_at	PDCD1	217190_x_at	ESR1	200023_s_at	EIF3F	232914_s_at	SYTL2	229538_s_at	IQGAP3
223834_at	CD274	211233_x_at	ESR1	215157_x_at	PABPC1	212510_at	GPD1L	208165_s_at	PRSS16
220049_s_at	PDCD1LG2	215104_at	NRIP2	228256_s_at	EPB41L4A	227733_at	TMEM63C	226189_at	ITGB8
222835_at	THSD4	229377_at	GRTP1	205990_s_at	WNT5A	235020_at	TAF4B	212998_x_at	HLA-DQB1
228708_at	RAB27B	244264_at	KLRG2	226192_at	AR	209138_x_at	IGLC1	215536_at	HLA-DQB2
209505_at	NR2F1	232179_at	LOC158863	204014_at	DUSP4	225973_at	TAP2	204149_s_at	GSTM4
226553_at	TMPRSS2	236390_at	SLX4IP	203963_at	CA12	223307_at	CDCA3	214123_s_at	NOP14-AS1
		213823_at	HOXA11			232001_at	PRKCQ-AS1		

^a^ Gene probes were derived from Affi-matrix microarray GE

*IM* immumodulatory, *MSL* mesenchymal stem like, *M* mesenchymal, *LAR* luminal androgen receptor, *BL* basal-like

Any possible contamination of the various PCR components was excluded by performing a PCR reaction with these components in the absence of the RT product for each set of experiments (contemplate control, NTC). For the statistical comparisons, the relative expression level of the mRNA of each specific gene was normalized against the amount of *GAPD* mRNA in the same RNA extract. All samples were analyzed in triplicate.

### Statistic analysis

Data are expressed as mean ± SEM. Differences between groups were identified by repeatedly measured one-way ANOVA, followed by Dunnet’s post hoc test. Differences between different groups were identified by Mann–Whitney *U* test for nonparametric analysis or the Student’s *t* test. A *p* value of <0.05 is considered statistically significant.

## Results

### Dataset collection and TNBC identification by bimodal filtering

**Fig. 1:**
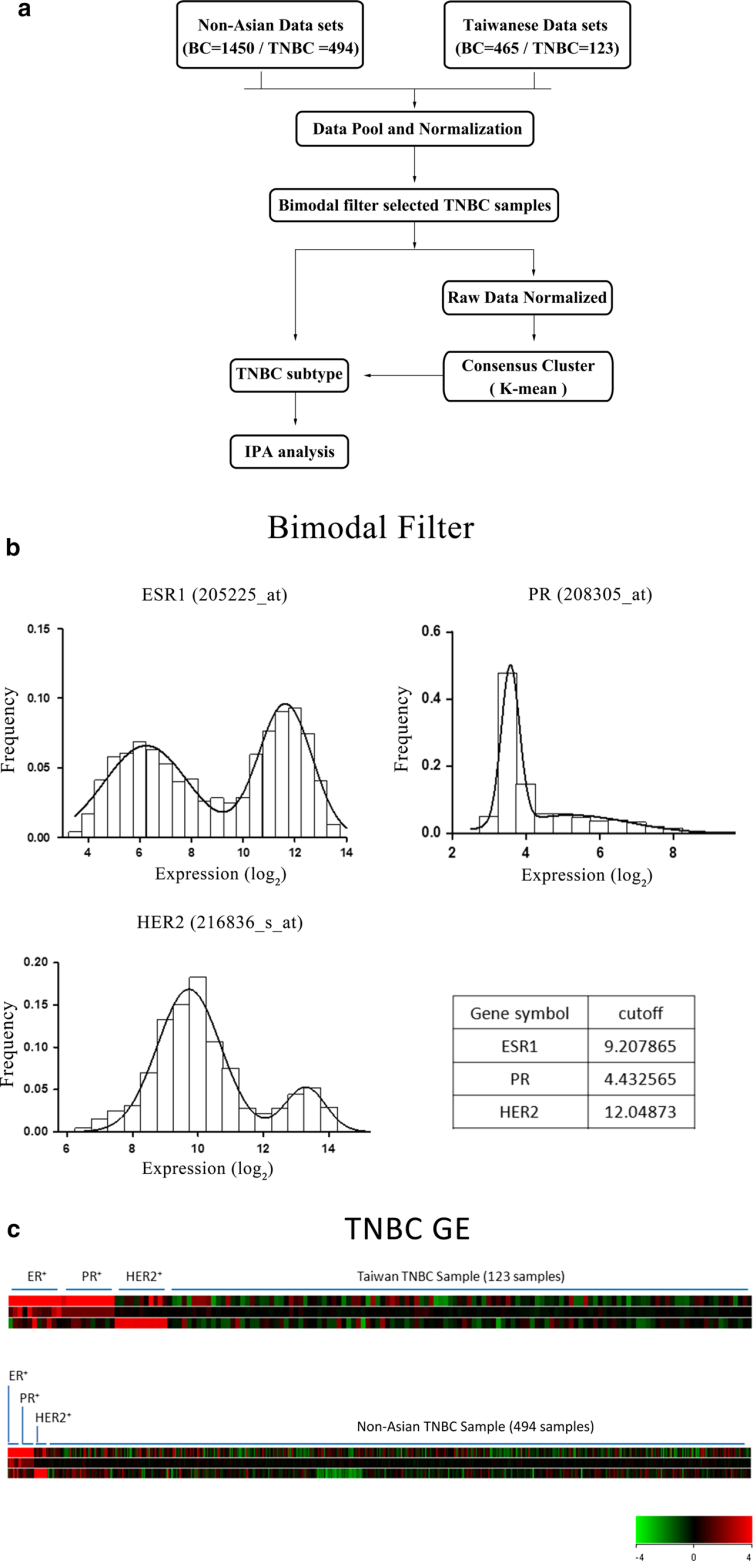
Protocol for the acquisition and analysis of the gene expression datasets. GEO Datasets for nonunion (12 groups, *n* = 1450) and Taiwanese (3 groups, *n* = 465) female breast cancer samples, including 617 triple-negative breast cancer (TNBC) samples, were acquired, normalized, and cluster analyzed (**a**). TNBC was identified by bimodal filtering (**b**) and was demonstrated in (**c**)

From June 2013 to September 2015, 57 patients whose tumor samples were screened as TNBC by immunohistochemistry (ER < 1%, PR < 1%, HER2, not amplified) were identified at Taipei Veterans General Hospital. These tumor samples were sent for microarray analysis. Next, two Taiwanese (*n* = 408) and twelve nonunion datasets (*n* = 1450) were downloaded from the public domain. Thus, a total of 1915 human breast cancer samples, including ours (*n* = 57), were available for expression analysis. The gene expression information generated from Affymetrix microarrays were then normalized independently using RMA procedures (Fig. 1a and Supplementary Reference 1).

The gene expression distributions of ER, PR, and HER2 for the TNBC samples were validated by two-component Gaussian distribution, and the cutoff point was estimated by maximum likelihood optimization using the optimize function (R statistical software) (Fig. 1b). This resulted in a heat map showing the TNBC tumors normalized along with positive controls for ER, PR, and HER2 (Fig. 1c). Finally, the TNBCs identified as true TNBCs (*n* = 617) were enrolled into the compiled dataset.

### The GE TNBC subtype samples of nonunion and Taiwanese women clustered in terms of the published 6-subtype gene lists

**Fig. 2:**
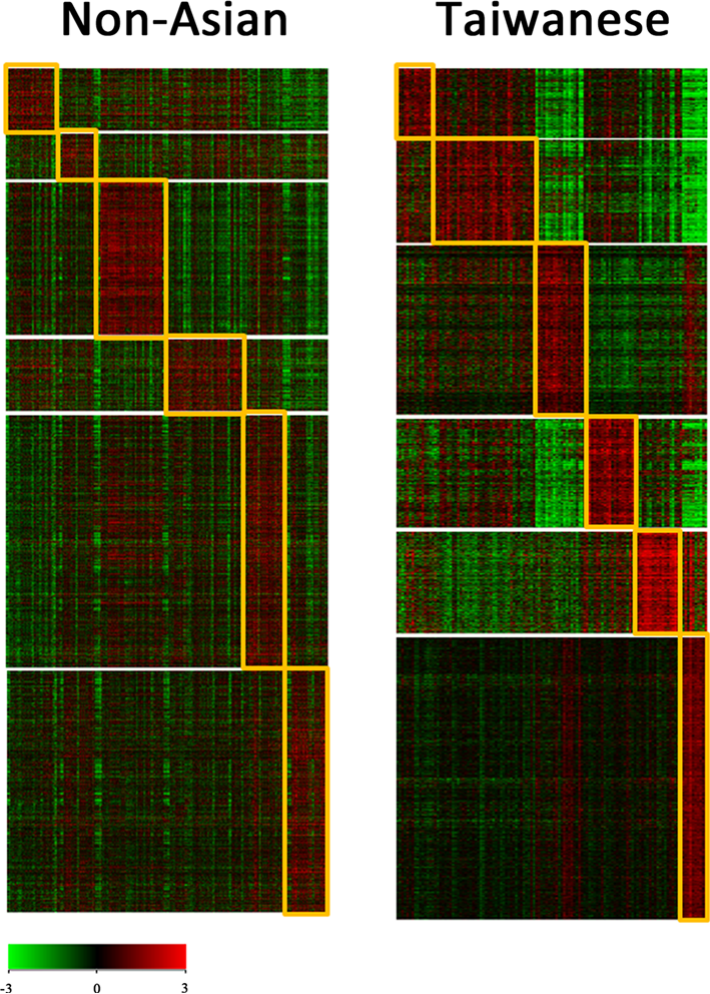
Heat maps of the clustered triple-negative breast cancer (TNBC) subtype for nonunion and Taiwanese women. The published gene lists of the six subtypes of TNBC were imported and used for the clustering of our compiled dataset, which consisted of a nonunion group (*left panel*) and a Taiwanese group (*right panel*) TNBC

Since TNBC subtyping has been suggested as a useful approach, we acquired the published gene lists of the 6-subtype of TNBC and used these for clustering of our compiled dataset, which included nonunion (Fig. 2, left panel) and Taiwanese (Fig. 2, right panel) women. The results showed that the percentages of TNBC subtypes in nonunion women, namely, BL1, BL2, IM, M, MSL, and LAR were 13.56, 8.91, 16.80, 20.45, 8.30, and 11.13%, respectively, while those in Taiwanese women was 14.63, 4.07, 17.89, 16.26, 17.89, and 20.33%, respectively.

**Fig. 3:**
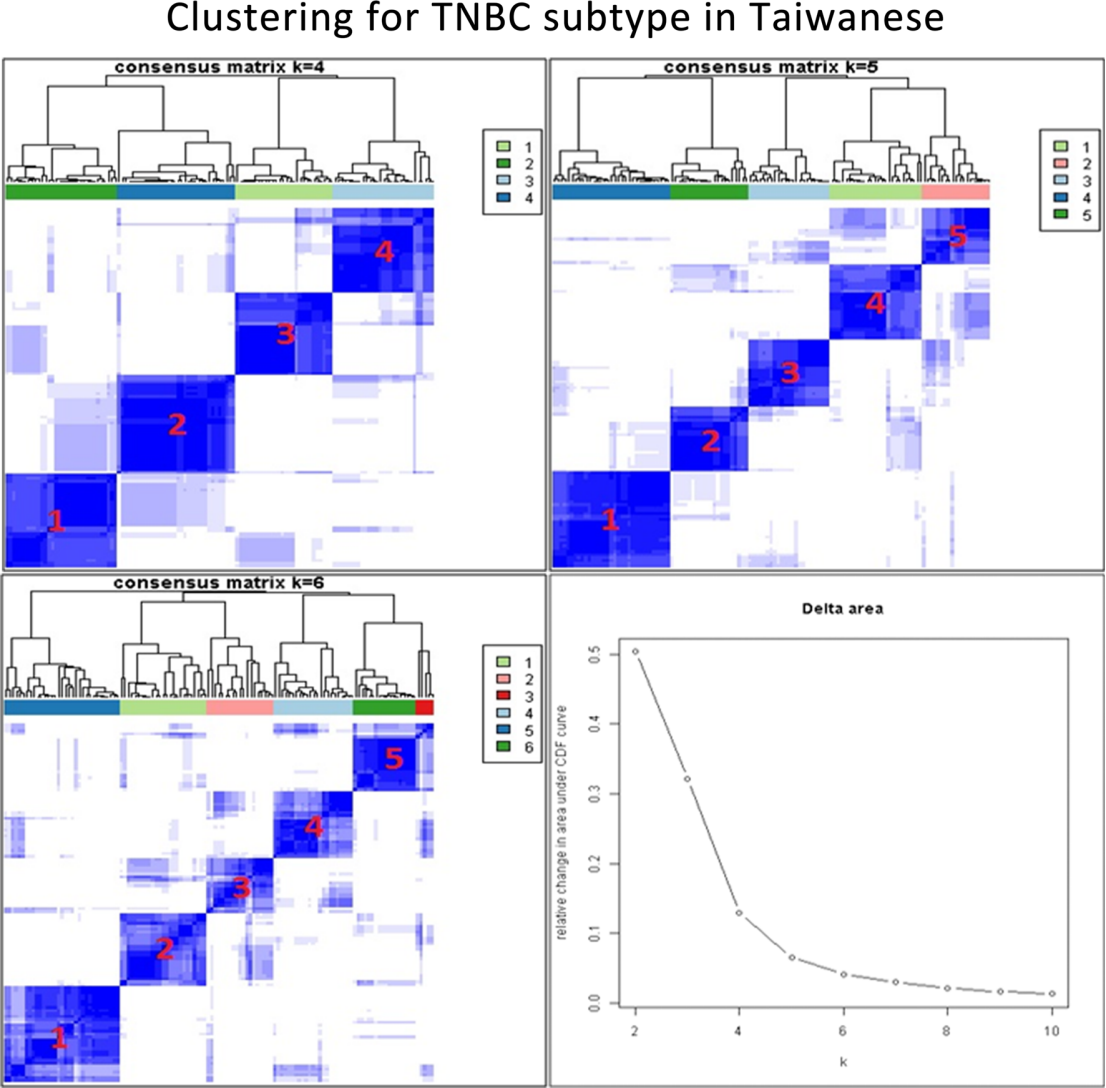
Cluster analysis of the triple-negative breast cancer (TNBC) subtype for Taiwanese women. After background correction of the Taiwanese data, consensus clustering and k-means clustering were used to determine the optimal number of TNBC subtypes. The optimal number of clusters was determined from the Consensus Cumulative Distribution Function (CDF)

**Fig. 4:**
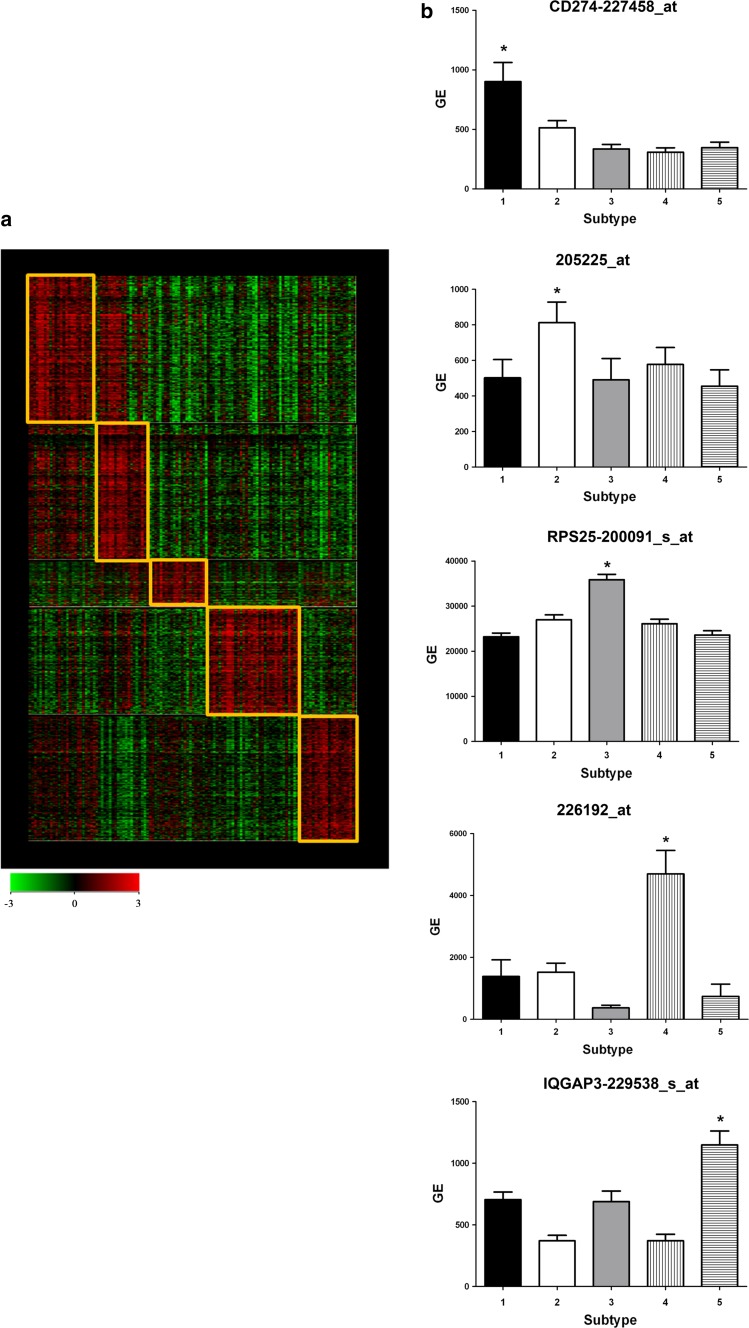
The triple-negative breast cancer (TNBC) subtypes for TNBC from Taiwanese women. The heat map shows five stable TNBC subtypes (**a**). The genes specific to each subtype are 274227458_at (*CD 274* or *PDL1*) for IM, 205225_at for MSL, 200091_s_at for M, 226192_at (androgen receptor) for LAR, and 229538_s_at (*IQGAP3*) for BL (**b**)

When the two groups of women are compared, there exist some discrepancies between nonunion and Taiwanese women in terms of TNBC subtypes. To address this, background correction for the Taiwanese data was performed and consensus clustering and *k*-means clustering were used to determine the optimal number of TNBC subtypes for Taiwanese (Fig. 3). The results showed that five stable subtypes were obtained based on the Taiwanese TNBC data (Fig. 4a). These were IM (13.82%), MSL (30.89%), M (22.76%), LAR (23.58%) and BL (8.94%). The genes specific to each subtype were 274227458_at (*CD 274* or *PDL1*) for IM, 205225_at for MSL, 200091_s_at for M, 226192_at (androgen receptor) for LAR, and 229538_s_at (*IQGAP3*) for BL (Fig. 4b). The genes specific to each TNBC subtype having been identified (Supplementary Reference 2) and correlated with the Lehmann et al. genes (Table 3) were analyzed using ingenuity pathway analysis (IPA); furthermore, their top canonic pathways, their upstream regulators, their top disease and their biofunctions were also analyzed. The results are summarized in Tables 4 and 5.

**Table 3:** Correlation of subtype-specific genes between Taiwanese’s and Lehmann’s genes

Lehmann’s subtypes	Present study				
	Subtype1 (IM)	Subtype2 (MSL)	Subtype3 (M)	Subtype4 (LAR)	Subtype5 (BL)
BL1	19.23^a^	0.00	8.70	0.00	50.00
BL2	0.00	5.26	8.70	3.03	4.55
IM	53.85	26.32	13.04	0.00	0.00
M	19.23	5.26	34.78	9.09	13.64
MSL	0.00	52.63	13.04	24.24	4.55
LAR	3.85	10.53	0.00	63.64	4.55

^a^ Data were presented as percentage (%)

*IM* immumodulatory, *MSL* mesenchymal stem like, *M* mesenchymal, *LAR* luminal androgen receptor, *BL* basal-like

**Table 4:** Ingenuity pathway analysis for up-regulated genes in TNBC subtypes

Name	*p*-value
Subtype 01 (immunomodulatory)	
Top canonical pathways	
CD28 signaling in T helper cells	7.02E−17
iCOS-iCOSL signaling in T helper cells	1.24E−16
Natural killer cell signaling	9.45E−15
Role of NFAT in regulation of the immune response	2.08E−13
T cell receptor signaling	3.69E−13
Top upstream regulators	
E2F4/IRF7/IRF1/E2F1/ESR1	
Top diseases and bio functions	
Cancer/organismal injury and abnormalities/gastrointestinal disease/infectious diseases/hematological disease	
Subtype 02 (mesenchymal stem like)	
Top canonical pathways	
EIF2 signaling	1.19E−17
iCOS-iCOSL signaling in T helper cells	1.15E−14
Hepatic fibrosis/hepatic stellate cell activation	1.27E−14
Crosstalk between dendritic cells and natural killer cells	3.20E−14
Tec kinase signaling	2.89E−12
Top upstream regulators	
CREBBP/MYCN/EP300/ID2/BCL6	
Top diseases and bio functions	
Cancer/organismal injury and abnormalities/inflammatory response/connective tissue disorders/skeletal and muscular disorders	
Subtype 03 (mesenchymal)	
Top canonical pathways	
EIF2 signaling	3.18E−69
Regulation of eIF4 and p70S6K signaling	2.92E−23
Oxidative phosphorylation	4.38E−18
mTOR signaling	1.45E−16
Mitochondrial dysfunction	5.81E−14
Top upstream regulators	
MYCN/MYC/HNF4A/DOT1L/HSF1	
Top diseases and bio functions	
Cardiovascular disease/developmental disorder/hereditary disorder/organismal injury and abnormalities	
Subtype 04 (luminal androgen receptor)	
Top canonical pathways	
NRF2-mediated oxidative stress response	8.86E−08
Xenobiotic metabolism signaling	3.00E−06
LPS/IL-1 mediated Inhibition of RXR function	1.64E−05
HIPPO signaling	5.69E−05
Clathrin-mediated endocytosis signaling	7.13E−05
Top upstream regulators	
ESR1/HNF4A/TP53/PGR/ESR2	
Top diseases and bio functions	
Cancer/organismal injury and abnormalities/gastrointestinal disease/hepatic system disease/developmental disorder	
Subtype 05 (basal-like)	
Top canonical pathways	
Role of BRCA1 in DNA damage response	2.92E−15
Hereditary breast cancer signaling	4.71E−14
Cell cycle: G2/M DNA damage checkpoint regulation	2.32E−13
Role of CHK proteins in cell cycle checkpoint control	4.39E−13
Mitotic roles of polo-like kinase	2.82E−12
Top upstream regulators	
E2F4/HNF4A/NUPR1/E2F1/ESR1	
Top diseases and bio functions	
Cancer/organismal injury and abnormalities/gastrointestinal disease/infectious diseases/hepatic system disease

**Table 5:** Ingenuity pathway analysis for down-regulated genes in TNBC subtypes

Name	*p*-value
Subtype 01 (immunomodulatory)	
Top canonical pathways	
EIF2 signaling	6.04E−25
Regulation of eIF4 and p70S6K signaling	2.40E−11
mTOR signaling	9.41E−10
Mitochondrial dysfunction	3.25E−08
Tight junction signaling	2.08E−07
Top upstream regulators	
MYCN/ESR1/HNF4A/CREB1/PGR	
Top diseases and bio functions	
Cancer/organismal injury and abnormalities/neurological disease/psychological disorders/gastrointestinal disease	
Subtype 02 (mesenchymal stem like)	
Top canonical pathways	
Protein ubiquitination pathway	4.08E−20
Role of CHK proteins in cell cycle checkpoint control	1.19E−13
Mitotic roles of polo-like kinase	1.45E−12
Hypoxia signaling in the cardiovascular system	1.14E−10
Role of BRCA1 in DNA damage response	1.29E−09
Top upstream regulators	
HNF4A/E2F4/ESR1/TP53/NUPR1	
Top diseases and bio functions	
Cancer/organismal injury and abnormalities/gastrointestinal disease/infectious diseases/hepatic system disease	
Subtype 03 (mesenchymal)	
Top canonical pathways	
B cell receptor signaling	2.16E−18
Leukocyte extravasation signaling	7.44E−16
Integrin signaling	5.48E−15
Molecular mechanisms of cancer	1.05E−14
Hepatic fibrosis/hepatic stellate cell activation	4.83E−12
Top upstream regulators	
ESR1/HNF4A/TP53/ERG/NR3C1	
Top diseases and bio functions	
Cancer/organismal injury and abnormalities/gastrointestinal disease/hepatic system disease/reproductive system disease	
Subtype 04 (luminal androgen receptor)	
Top canonical pathways	
Role of BRCA1 in DNA damage response	3.35E−14
Molecular mechanisms of cancer	4.43E−11
Hereditary breast cancer signaling	1.03E−10
Crosstalk between dendritic cells and natural killer cells	2.98E−10
Natural killer cell signaling	6.01E−10
Top upstream regulators	
E2F4/IRF7/E2F1/CDKN2A/IRF1	
Top diseases and bio functions	
Cancer/organismal injury and abnormalities/gastrointestinal disease/infectious diseases/hematological disease	
Subtype 05 (basal-like)	
Top canonical pathways	
EIF2 signaling	3.99E−20
Hepatic fibrosis/hepatic stellate cell activation	1.52E−17
Crosstalk between dendritic cells and natural killer cells	1.58E−12
Primary immunodeficiency signaling	1.13E−09
LPS/IL-1 mediated inhibition of RXR function	1.91E−09
Top upstream regulators	
MYCN/CREBBP/EP300/SMARCA4/CTNNB1	
Top diseases and bio functions	
Cancer/organismal injury and abnormalities/dermatological diseases and conditions/connective tissue disorders/inflammatory disease	

### Model identification using representative genes in human TNBC cell lines

Using the gene lists selected from the clustering results (Supplementary Reference 2), which were identified as specific to each subtype, real-time PCR was carried targeting a 47 gene signature (Table 2) using customized chip. This analysis was carried out on five human TNBC cell lines, namely, MDA-MB-468 (BL1), MDA-MB-231 (MSL), BT-549 (M), MDA-MB-453 (LAR), and DU4475 (IM).

**Fig. 5:**
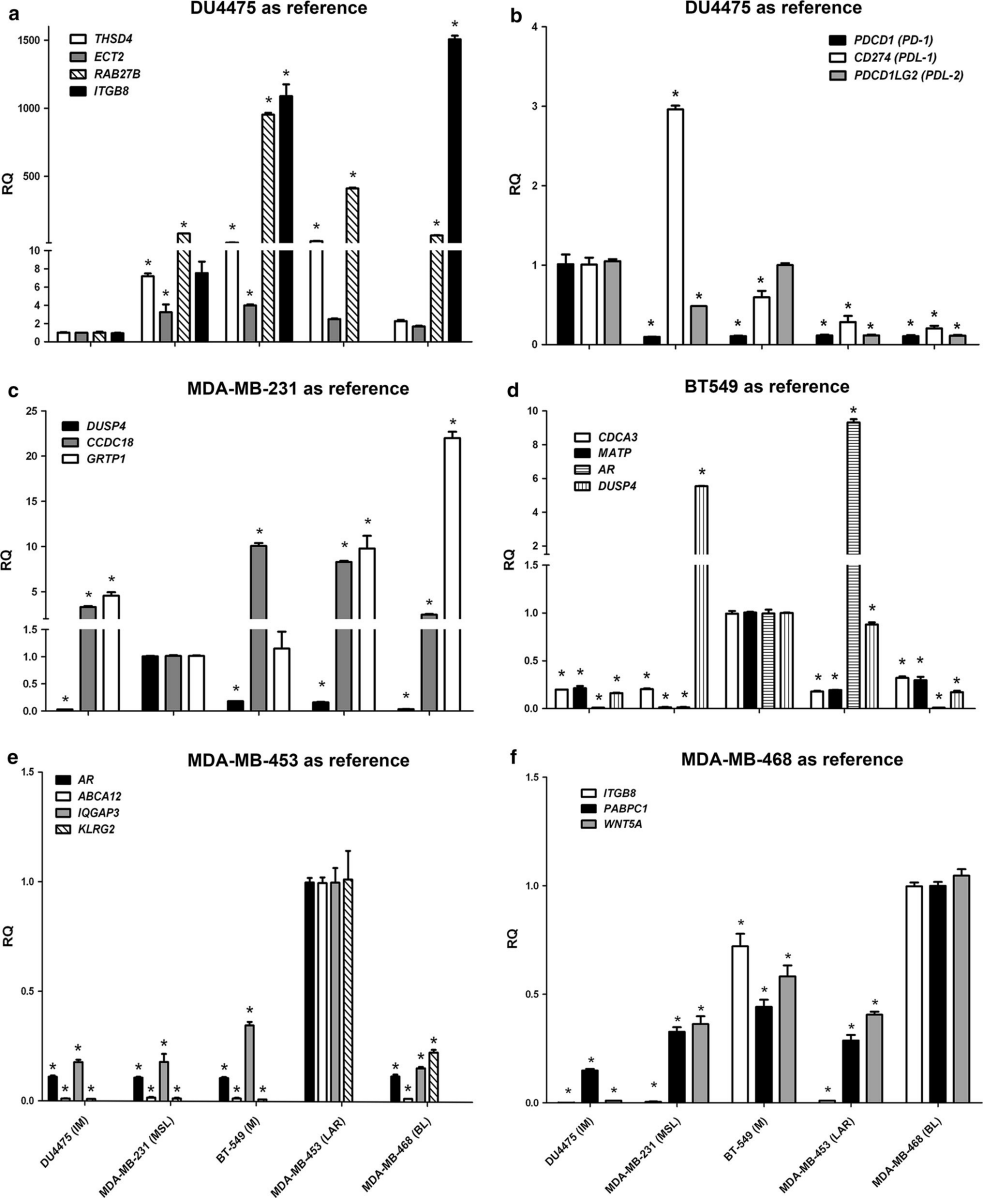
Model identification using representative genes in human triple-negative breast cancer (TNBC) cell lines. Using the DU4475 (IM) as the reference line, there was significant downregulation of *THSD4*, *ECT2*, *RAB27B*, *and ITGB8* (**a**) together with significant upregulation of *PDCD1* (*PD1*), *CD274* (*PDL1*) (except MDAMB231), and *PDCD1LG2* (*PDL2*) (**b**) compared to the other cell lines).Using the MDA-MB-231 (MSL) (**c**) as the reference line, there was significant upregulation of *DUSP4* together with significant downregulation of *CCDC18* and *GRTP1* compared to other cell lines. Using the BT-549 (M) (**d**) as the reference line, there was significant upregulation of *CDCA3* and *MATP* in this line, compared to other cell lines and there was significant upregulation of *DUSP4* in MDA-MB-231 (MSL) and AR in MDA-MB-453 (LAR), compared to the BT-549 (M) line. Using the MDA-MB-453 (LAR) as reference line (**e**), there was significant upregulation of *AR*, *ABCA12*, *IQGAP3*, and *KLRG2* in this line, compared to other cell lines. Using the MDA-MB-468 (BL1) as the reference line (**f**), there was significant upregulation of *TPGB8*, *PABPC1, *and *WNT5A* in this line, compared to other cell lines

Using DU4475 (IM) as the reference line, significant downregulation of *THSD4*, *ECT2*, *RAB27B*, and *ITGB8* was found (Fig. 5a), together with significant upregulation of *PDCD1* (*PD1*), *CD274* (*PDL1*) (except MDAMB231), and *PDCD1LG2* (*PDL2*) (Fig. 5b), in DU4475 compared to the other cell lines MDA-MB-468 (BL1), MDA-MB-231 (MSL), BT-549 (M), and MDA-MB-453 (LAR). Using MDA-MB-231 (MSL) as the reference line, significant upregulation of *DUSP4*, together with significant downregulation of *CCDC18* and *GRTP1* (Fig. 5c) were found in MDA-MB-231 compared to the other cell lines. Using BT-549 (M) as the reference line, significant upregulation of *CDCA3* and *MATP* in BT-549 (Fig. 5d) was found compared to the other cell lines. However, in addition these findings for BT-549, it needs to be noted that there was significant upregulation of *DUSP4* in MDA-MB-231 (MSL) and of *AR* in MDA-MB-453 (LAR) compared to BT-549 (M) (Fig. 5d). When using MDA-MB-453 (LAR) as the reference line, significant upregulation of *AR*, *ABCA12*, *IGQAP3*, and *KLRG2* in MDA-MB-453 (Fig. 5e) was found. Finally, when using MDA-MB-468 (BL1) as the reference line, significant upregulation of *ITGB8*, *PABPC1, *and *WNT5A* in MDA-MB-468 (Fig. 5f) was found.

## Discussion

Breast cancer raises important health problem worldwide. Even after considering the many therapies for the various subtypes of breast cancer, treatment of triple-negative breast cancer (TNBC) remains a challenging issue. The heterogeneity of TNBC tumors contributes to their poor response to chemotherapy, and this had led to the development of TNBC subtyping. In this study, we compiled GE profiles from publically available breast cancer microarray datasets that included both nonunion and Taiwanese populations. These were then cluster analyzed, which was followed by model identification using representative genes in TNBC cell lines.

There is consensus that significant preprocessing, including background adjustment, normalization, and summarization, is required before a specific gene may be accurately assessed using a complied dataset [29]. Based on the published gene lists of the six subtypes of TNBC proposed by Lehmann et al. [7], using our compiled dataset, we found that there was clearly distinct subtype presentation among nonunion samples (Fig. 2, left panel), but this subtyping was not the same for the Taiwanese population (Fig. 2, right panel). Based on these finding, we renormalized the Taiwanese data using the MAS5 procedure and carried out clustering; this resulted in five rather than six clear subtypes being present in the Taiwanese population. Previous studies have suggested that the GCRMA approach might be responsible for introducing artifacts into the data analysis and that this can lead to a systematic overestimate of pairwise correlations within the data. In this context, it has been suggested that the MAS5 approach provides the most faithful cellular network reconstruction [30, 31].

Although from three to six TNBC subtypes have been proposed by various authors either using gene ontologies [10, 32], therapeutic targets [11, 12] or mRNA profiles as the diagnostic criteria [13], the exact number of TNBC subtypes that occur in women remains an open question [14]. Our findings identified five subtypes and these were the IM, MSL, M, LAR and BL subtypes. Interestingly, the BL1 and BL2 subtypes of the Lehmann’s six type classification were clustered as a single BL subtype in our Taiwanese dataset. We attribute this discrepancy to a result of a smaller sample size, as the number of subtypes tends to increase with sample size.

Several lines of evidence suggest that the interactions of cancer cells with their microenvironment are a critical feature during tumor progression. The cell types involved in such interactions are not necessarily stromal cells [33], but also include macrophages [34], endothelial cells [35], and T cells [36]. Interestingly, we found significant upregulation of *PDCD1* (*PD1)*, *CD274* (*PDL1*), and *PDCD1LG2* (*PDL2*) expression in the IM subtype compared to the MSL subtype in our compiled dataset. However, when using DU4475 (IM) as the reference line, there was significant upregulation of *PDCD1* (*PD1*), and *PDCD1LG2* (*PDL2*), but not of *CD274* (*PDL1*), compared to MDA-MB-231 (MSL) (Supplementary Reference 3). We attribute this discrepancy to the study samples used, namely, cell lines versus tumor tissue. In the former, only cancer cells were investigated, while in the latter, cancer cells and other cells participating in the tumor microenvironment were investigated as a pool. It should be noted that the IM and MSL subtypes in our dataset share many canonical pathways, such as the iCOS-iCOSL signaling pathway (Table 4), which suggests the presence of significant similarity between these two subtypes. This seems to be supported by previous findings, which indicated that some transcripts present in the IM and MSL subtypes are contributed to by the tumor microenvironment [14].

The expression of the androgen receptor (AR) plays various different prognostic roles depending on the breast cancer subtype, such as the difference between ER-positive and ER-negative breast cancers with the expression levels of around 67–88% [37, 38] and 12–50% [39] for AR, respectively. Importantly in this context, it should be noted that the prevalence of AR expression has been found to range from 0–53% of TNBC [40].

In our compiled dataset, the percentages of the LAR subtype among nonunion and Taiwanese TNBC women were found to be 11.13 and 23.58%, respectively. There is evidence suggesting that AR expression is about 60% among early breast cancers and is more frequently expressed in ER-positive than ER-negative breast cancers [41]. We speculate that ethnic differences might explain the variation in the percentage of the AR subtype between these different populations. However, further validation of this speculation is needed. If we examine cell line-specific gene expression, although the AR gene in BT-549 (M) is upregulated compared to DU4475 (IM), MDA-MB-468 (BL1) and MDA-MB-231 (MSL), the AR gene transcript in MDA-MB-453 (LAR) is ninefold higher than in BT-549 (M), which suggests that this change in AR gene expression is specific to the LAR subtype. Recent discrepancies concerning the role of AR have been noted in various TNBC basic and clinical studies and both AR agonist and AR antagonist clinical trials have been designed for the treatment of TNBC and ER^+^ breast cancers [41–43]. Thus, the therapeutic role of AR remains an open question.

In summary, our findings suggest that there exist different presentations between nonunion and Taiwanese female populations in terms of TNBC subtypes. The fact that there seems to be correlation between the IM and MSL subtypes suggests the involvement of the tumor microenvironment in TNBC subtype classification might help to provide important information when selecting therapeutic targets or designing for clinical trials for TNBC patients.

## Electronic supplementary material

Below is the link to the electronic supplementary material.

Supplementary material 1 (PDF 111 kb)

Supplementary material 2 (PDF 113 kb)

Supplementary material 3 (PDF 219 kb)

## Abbreviations

BL: Basal-like
CDF: Cumulative Distribution Function
ER: Estrogen receptor
GE: Gene expression
HER2: Human epidermal growth receptor 2
IM: Immune-modulatory
M: Mesenchymal
MSL: Mesenchymal stem cell like
LAR: Luminal androgen receptor
PR: Progenstin receptor
TNBC: Triple-negative breast cancer
