# Supplementary material for: A comparison of the molecular subtypes of triple-negative breast cancer among non-Asian and Taiwanese women
Source: Breast Cancer Res Treat. 2017 Mar 15;163(2):241–54. doi: 10.1007/s10549-017-4195-7 (PMC5410215; doi:10.1007/s10549-017-4195-7)
Supplement: Supplementary file 1 — Supplementary material 1 (PDF 111 kb) [file 10549_2017_4195_MOESM1_ESM.pdf]

GSE95700	Molecular
Taiwanese	
GSM2522296	BRCA01
GSM2522297	BRCA02
GSM2522298	BRCA03
GSM2522299	BRCA04
GSM2522300	BRCA05
GSM2522301	BRCA06
GSM2522302	BRCA07
GSM2522303	BRCA08
GSM2522304	BRCA09
GSM2522305	BRCA10
GSM2522306	BRCA11
GSM2522307	BRCA12
GSM2522308	BRCA13
GSM2522309	BRCA14
GSM2522310	BRCA15
GSM2522311	BRCA16
GSM2522312	BRCA17
GSM2522313	BRCA18
GSM2522314	BRCA19
GSM2522315	BRCA20
GSM2522316	BRCA21
GSM2522317	BRCA22
GSM2522318	BRCA23
GSM2522319	BRCA24
GSM2522320	BRCA25
GSM2522321	BRCA26
GSM2522322	BRCA27
GSM2522323	BRCA28
GSM2522324	BRCA29
GSM2522325	BRCA30
GSM2522326	BRCA31
GSM2522327	BRCA32
GSM2522328	BRCA33
GSM2522329	BRCA34
GSM2522330	BRCA35
GSM2522331	BRCA36
GSM2522332	BRCA37
GSM2522333	BRCA38
GSM2522334	BRCA39
GSM2522335	BRCA40
GSM2522336	BRCA41

GSM2522337	BRCA42
GSM2522338	BRCA43
GSM2522339	BRCA44
GSM2522340	BRCA45
GSM2522341	BRCA46
GSM2522342	BRCA47
GSM2522343	BRCA48
GSM2522344	BRCA49
GSM2522345	BRCA50
GSM2522346	BRCA51
GSM2522347	BRCA52
GSM2522348	BRCA53
GSM2522349	BRCA54
GSM2522350	BRCA55
GSM2522351	BRCA56
GSM2522352	BRCA57
