# Supplementary material for: A comparison of the molecular subtypes of triple-negative breast cancer among non-Asian and Taiwanese women
Source: Breast Cancer Res Treat. 2017 Mar 15;163(2):241–54. doi: 10.1007/s10549-017-4195-7 (PMC5410215; doi:10.1007/s10549-017-4195-7)
Supplement: Supplementary file 2 — Supplementary material 2 (PDF 113 kb) [file 10549_2017_4195_MOESM2_ESM.pdf]

Supplementary reference 2

Those genes specific to each TNBC subtype were defined as followings:

1) The strongest probe with fold change (ratio) >1.75(up-regulated) or <0.5(down-regulated), compared with other subtypes;
2) percentage of sample with GE difference >0 (sample GE – mean GE of other subtypes) >80%; and
3) a p value < 10^{-4} (t-test : specific subtype vs other subtype).

Subtype 01 (IM) UP

probe	P value	ratio	against	Gene
231577_s_at	6.29997E-07	2.549248	96.15385	GBP1
206513_at	3.1598E-06	2.048115	88.46154	AIM2
237753_at	3.75677E-06	1.935923	84.61538	IL21R
232362_at	1.71582E-05	1.784329	92.30769	CCDC18
202307_s_at	1.96534E-05	2.131318	84.61538	TAP1
206486_at	3.02016E-05	2.19851	88.46154	LAG3
223887_at	3.0478E-05	2.33936	80.76923	GPR132
226603_at	3.46741E-05	1.975948	84.61538	SAMD9L
206082_at	4.77363E-05	1.82896	84.61538	HCP5
224701_at	6.51894E-05	1.874285	80.76923	PARP14
228439_at	8.32161E-05	1.786656	80.76923	BATF2
206715_at	8.92339E-05	1.885128	88.46154	TFEC
1557267_s_at	9.98109E-05	2.095467	80.76923	LOC284952

Subtype 01 (IM) Down

Probe	P value	ratio	against	Gene
219093_at	1.26E-07	0.47301	96.15385	PID1
236359_at	2.99E-07	0.423393	96.15385	SCN4B
219719_at	1.93E-06	0.356835	92.30769	HIGD1B
211734_s_at	1.97E-06	0.304885	100	FCER1A
230889_at	2.19E-06	0.435712	96.15385	LOC645321
214418_at	2.49E-06	0.434995	88.46154	CT62
222835_at	2.57E-06	0.488464	96.15385	THSD4
222722_at	7.57E-06	0.310657	96.15385	OGN
1561101_at	1.01E-05	0.469398	92.30769	LOC153469
203951_at	1.11E-05	0.463138	88.46154	CNN1
205258_at	2.05E-05	0.403753	100	INHBB
203453_at	2.38E-05	0.397197	88.46154	SCNN1A
228708_at	3.5E-05	0.490707	84.61538	RAB27B
244472_at	4.71E-05	0.430454	88.46154	LOC388630
209505_at	5.08E-05	0.424527	96.15385	NR2F1
202016_at	5.15E-05	0.433857	88.46154	MEST
1557446_x_at	5.61E-05	0.450701	88.46154	TREML3
226553_at	5.94E-05	0.394853	84.61538	TMPRSS2
236351_at	9.77E-05	0.358496	92.30769	LOC389023

Subtype 02 (MSL) up

Probe	P value	ratio	against	Gene
227427_at	2.48E-07	1.989884	100	ARHGEF25
206485_at	9.89E-07	1.99183	100	CD5
206437_at	1.23E-06	2.101434	89.47368	S1PR4
217767_at	3.35E-06	1.86509	94.73684	C3
227915_at	3.63E-06	2.666264	84.21053	ASB2
1555298_a_at	5.41E-06	1.761484	89.47368	VWA3B
210884_s_at	1.34E-05	1.783177	94.73684	SPAG11A
208321_s_at	1.6E-05	2.488834	89.47368	CABP1
227030_at	1.7E-05	2.47327	89.47368	IKZF3
213958_at	1.88E-05	1.76413	84.21053	CD6
215104_at	2.38E-05	1.968474	84.21053	NRIP2
240890_at	2.44E-05	1.901846	89.47368	LOC643733
232821_at	2.64E-05	1.771896	84.21053	GTSF1L
213990_s_at	2.99E-05	2.386412	84.21053	PAK7
206121_at	4.65E-05	2.597928	94.73684	AMPD1
220581_at	4.74E-05	1.797017	84.21053	C6orf97
209770_at	5.36E-05	1.853422	84.21053	BTN3A1
211062_s_at	7.44E-05	2.282405	89.47368	CPZ
241049_at	7.87E-05	1.750072	89.47368	GRM7
221529_s_at	7.97E-05	1.83872	89.47368	PLVAP
225373_at	9.48E-05	1.750317	89.47368	C10orf54

Subtype 02 (MSL) down

Probe	P value	ratio	against	Gene
202381_at	4.53E-14	0.446104	100	ADAM9
218239_s_at	1.84E-13	0.486239	100	GTPBP4
1558152_at	1.21E-11	0.368682	100	LOC100131262
221922_at	2.15E-10	0.424204	100	GPSM2
229377_at	1.15E-09	0.450115	100	GRTP1
221539_at	3.25E-09	0.441437	100	EIF4EBP1
215071_s_at	9.35E-09	0.464779	100	HIST1H2AC
202095_s_at	1.22E-08	0.382769	94.73684	BIRC5
244264_at	2.37E-08	0.417499	100	KLRG2
226319_s_at	3.9E-08	0.428177	100	THOC4
222608_s_at	7.46E-08	0.404062	94.73684	ANLN
226661_at	1.29E-07	0.384637	94.73684	CDCA2
232179_at	1.41E-07	0.45334	100	LOC158863
214710_s_at	2.79E-07	0.491761	94.73684	CCNB1
1555409_a_at	4.68E-07	0.293408	89.47368	BAGE2
236390_at	6.82E-07	0.33243	94.73684	C20orf94
209369_at	2.38E-06	0.496992	94.73684	ANXA3
201563_at	2.73E-06	0.33271	94.73684	SORD
202712_s_at	7.04E-06	0.370185	94.73684	CKMT1A
229332_at	7.1E-06	0.415161	94.73684	HPDL
206307_s_at	8.9E-06	0.419937	89.47368	FOXD1
242350_s_at	1.07E-05	0.360764	89.47368	LOC100128098
204675_at	1.13E-05	0.426342	89.47368	SRD5A1
231192_at	1.38E-05	0.368047	100	LPAR3
236898_at	1.41E-05	0.318109	100	LOC100288781
205891_at	1.52E-05	0.49827	94.73684	ADORA2B
243389_at	2.95E-05	0.481427	94.73684	PRH1
206110_at	6.11E-05	0.408223	94.73684	HIST1H3H
213823_at	6.77E-05	0.287473	94.73684	HOXA11
1556194_a_at	7.88E-05	0.458483	94.73684	LOC100507455
234291_s_at	8.52E-05	0.493014	94.73684	SLC6A20
207383_s_at	8.71E-05	0.378425	89.47368	RHBDL1

Subtype 03 (M) up

Probe	P value	ratio	against	Gene
200091_s_at	8.11E-10	1.436542	100	RPS25
208834_x_at	3.55E-09	1.272887	100	RPL23A
210646_x_at	1.1E-08	1.373134	100	RPL13A
211927_x_at	3E-08	1.379296	100	EEF1G
202029_x_at	7.01E-08	1.46581	100	RPL38
208692_at	9.04E-08	1.392847	95.65217	RPS3
201268_at	9.92E-08	1.562372	91.30435	NME1-NME2
200025_s_at	1.96E-07	1.382503	95.65217	RPL27
221475_s_at	3.75E-07	1.450964	100	RPL15
213801_x_at	1.21E-06	1.381646	95.65217	RPSA
200705_s_at	1.36E-06	1.470506	100	EEF1B2
213564_x_at	2.46E-06	1.772632	86.95652	LDHB
AFFX-HUMGAPDH/M33197_3_at	2.52E-06	1.376094	82.6087	GAPDH
213583_x_at	3.08E-06	1.205685	95.65217	EEF1A1
222229_x_at	1.01E-05	1.568626	82.6087	RPL26
200936_at	2.1E-05	1.350008	86.95652	RPL8
200651_at	2.22E-05	1.408621	82.6087	GNB2L1
200023_s_at	2.34E-05	1.395884	86.95652	EIF3F
215157_x_at	2.47E-05	1.237558	86.95652	PABPC1
200089_s_at	3.01E-05	1.377741	82.6087	RPL4
212284_x_at	4.02E-05	1.171134	82.6087	TPT1

Subtype 03 (M) down

Probe	P value	ratio	against	Gene
228256_s_at	1.35E-12	0.42478	100	EPB41L4A
205990_s_at	1.53E-08	0.47207	100	WNT5A
220122_at	2.92E-08	0.485758	100	MCTP1
226192_at	7.51E-08	0.15685	100	AR
220894_x_at	4.19E-07	0.400334	91.30435	PRDM12
202203_s_at	4.78E-07	0.413448	91.30435	AMFR
228342_s_at	8.21E-07	0.493676	95.65217	ALPK3
211637_x_at	2.36E-06	0.201171	95.65217	IGHA1
206927_s_at	2.57E-06	0.481896	100	GUCY1A2
206155_at	2.63E-06	0.166914	100	ABCC2
1560853_x_at	2.8E-06	0.495236	91.30435	ZNF826P
205597_at	2.89E-06	0.143107	100	SLC44A4
244205_at	2.95E-06	0.490128	86.95652	ALAS2
219695_at	4.99E-06	0.498008	86.95652	SMPD3
226132_s_at	6.15E-06	0.448112	91.30435	MANEAL
223218_s_at	1.1E-05	0.497607	82.6087	NFKBIZ
1566833_x_at	1.48E-05	0.445395	86.95652	TOP1P2
210911_at	1.54E-05	0.490287	86.95652	ID2B
1555980_a_at	1.78E-05	0.431334	91.30435	FLJ39609
213228_at	2.21E-05	0.484359	95.65217	PDE8B
204014_at	2.47E-05	0.369098	91.30435	DUSP4
210652_s_at	2.61E-05	0.48505	86.95652	TTC39A
219956_at	2.74E-05	0.481951	91.30435	GALNT6
209071_s_at	2.87E-05	0.492017	91.30435	RGS5
221022_s_at	3.19E-05	0.483218	95.65217	PMFBP1
208411_x_at	6.54E-05	0.452963	91.30435	PPEF2
1567681_at	7.13E-05	0.468972	86.95652	SNORA74A
203963_at	8.12E-05	0.362816	100	CA12
1559065_a_at	8.78E-05	0.45756	82.6087	CLEC4G

Subtype 04 (LAR) up

Probe	P value	ratio	against	Gene
218211_s_at	5.28E-08	3.553376	81.81818	MLPH
216092_s_at	8.78E-08	2.095296	87.87879	SLC7A8
224990_at	3.37E-07	2.764749	81.81818	C4orf34
215465_at	3.97E-07	5.737718	84.84848	ABCA12
226992_at	4.64E-07	2.738339	87.87879	NOSTRIN
212458_at	9.4E-07	2.185052	90.90909	SPRED2
204137_at	1.02E-06	2.053697	96.9697	GPR137B
212181_s_at	1.37E-06	2.107925	87.87879	NUDT4
222209_s_at	1.5E-06	2.530285	84.84848	TMEM135
232914_s_at	2.51E-06	2.15907	84.84848	SYTL2
212510_at	3.05E-06	2.676407	84.84848	GPD1L
227733_at	3.22E-06	2.544072	81.81818	TMEM63C
224836_at	3.32E-06	2.058586	84.84848	TP53INP2
213012_at	3.62E-06	2.136718	87.87879	NEDD4
204573_at	4E-06	3.132224	87.87879	CROT
208788_at	4.58E-06	2.286128	84.84848	ELOVL5
201952_at	4.82E-06	3.530625	84.84848	ALCAM
201931_at	7.89E-06	2.051655	81.81818	ETFA
210886_x_at	1.07E-05	2.231649	90.90909	TP53TG1
205759_s_at	1.61E-05	2.415052	81.81818	SULT2B1
226192_at	1.78E-05	4.708602	81.81818	AR
1563022_at	2.15E-05	2.98726	81.81818	CCDC160
219087_at	2.38E-05	3.071985	81.81818	ASPN
227001_at	2.48E-05	2.209301	81.81818	NIPAL2
213506_at	2.82E-05	3.29242	90.90909	F2RL1
201596_x_at	5.76E-05	2.073944	81.81818	KRT18
209425_at	5.86E-05	2.183917	81.81818	AMACR
217681_at	6.57E-05	2.02109	81.81818	LOC100289775
204378_at	8.54E-05	3.124832	84.84848	BCAS1

Subtype 04 (LAR) down

Probe	P value	ratio	against	Gene
235020_at	6.94E-15	0.426822	100	TAF4B
221505_at	7.62E-14	0.374117	93.93939	ANP32E
205569_at	1.36E-13	0.28271	100	LAMP3
209842_at	1.84E-13	0.146988	93.93939	SOX10
209138_x_at	8.98E-13	0.458271	96.9697	IGL@
205044_at	2.01E-12	0.133245	96.9697	GABRP
209337_at	2.85E-12	0.465042	96.9697	PSIP1
219684_at	5.45E-12	0.339385	93.93939	RTP4
206513_at	6E-12	0.432285	100	AIM2
207979_s_at	6.28E-12	0.343892	96.9697	CD8B
204613_at	7.82E-12	0.460113	90.90909	PLCG2
229849_at	2.18E-11	0.354069	100	WIPF3
221016_s_at	2.67E-11	0.446628	93.93939	TCF7L1
228557_at	4.56E-11	0.382166	96.9697	L3MBTL4
225973_at	5E-11	0.44612	93.93939	TAP2
223307_at	7.42E-11	0.422136	96.9697	CDCA3
220150_s_at	9.05E-11	0.48019	93.93939	FAM184A
232001_at	1.18E-10	0.396134	100	LOC439949
202307_s_at	1.25E-10	0.408828	96.9697	TAP1
220059_at	2.64E-10	0.361037	100	STAP1
206364_at	2.78E-10	0.404446	96.9697	KIF14
206273_at	3.06E-10	0.442532	90.90909	SLMO1
227212_s_at	3.62E-10	0.446159	93.93939	PHF19
227285_at	4.59E-10	0.464921	93.93939	C1orf51
219863_at	4.96E-10	0.354196	100	HERC5
237753_at	6.72E-10	0.494398	96.9697	IL21R
210073_at	7.84E-10	0.355965	96.9697	ST8SIA1
203828_s_at	8.45E-10	0.362866	96.9697	IL32
226603_at	9.24E-10	0.451702	100	SAMD9L

Subtype 05 (BL)up

Probe	P value	ratio	against	Gene
219787_s_at	9.22E-08	1.851767	90.90909	ECT2
231984_at	2.41E-07	2.14251	95.45455	MTAP
203712_at	1.72E-06	1.85785	90.90909	KIAA0020
208694_at	4.38E-06	1.788473	90.90909	PRKDC
229538_s_at	1.08E-05	2.170404	90.90909	IQGAP3
235088_at	1.88E-05	1.807248	81.81818	C4orf46
213007_at	1.99E-05	1.827653	86.36364	FANCI
204641_at	2.04E-05	2.182492	90.90909	NEK2
208165_s_at	2.97E-05	2.177928	90.90909	PRSS16
226556_at	3.32E-05	1.857084	90.90909	MAP3K13
222036_s_at	3.61E-05	1.952045	86.36364	MCM4
233852_at	4.93E-05	1.844117	86.36364	POLH
226189_at	6.36E-05	1.862024	90.90909	ITGB8
242283_at	8.23E-05	1.889559	86.36364	DNAH14

Subtype 05 (MSL) down

Probe	P value	ratio	against	Gene
212998_x_at	1.79E-08	0.496589	95.45455	HLA-DQB1
222760_at	2.48E-07	0.477257	95.45455	ZNF703
211902_x_at	3.35E-07	0.444651	95.45455	TRD@
224403_at	3.61E-07	0.467267	100	FCRL4
227030_at	6.71E-07	0.347643	90.90909	IKZF3
211798_x_at	1.94E-06	0.215887	100	IGLJ3
220918_at	2.35E-06	0.386846	90.90909	C21orf96
215536_at	4.45E-06	0.494027	95.45455	HLA-DQB2
216576_x_at	4.68E-06	0.30743	95.45455	IGK@
230273_at	5.85E-06	0.472105	90.90909	C6orf165
226226_at	6.22E-06	0.167473	100	TMEM45B
219463_at	6.72E-06	0.453496	90.90909	C20orf103
215333_x_at	9.45E-06	0.364676	95.45455	GSTM1
243795_s_at	1.21E-05	0.468455	90.90909	LOC440900
1564591_a_at	1.25E-05	0.493976	90.90909	TMC1
1553883_at	1.87E-05	0.450813	95.45455	ZNF99
231240_at	2.49E-05	0.364479	95.45455	DIO2
1563904_at	2.89E-05	0.395324	86.36364	LOC100129620
1569729_a_at	3.17E-05	0.430985	90.90909	ASZ1
207635_s_at	3.5E-05	0.490911	86.36364	KCNH1
1553829_at	3.97E-05	0.439512	90.90909	C2orf58
242534_at	4.23E-05	0.435852	90.90909	LOC100288221
208471_at	4.65E-05	0.454571	86.36364	HPR
1562049_at	4.81E-05	0.386175	90.90909	LOC100287807
204149_s_at	4.85E-05	0.48522	86.36364	GSTM4
210643_at	5.32E-05	0.442089	95.45455	TNFSF11
237086_at	6.5E-05	0.201702	95.45455	FOXA1
214123_s_at	9.1E-05	0.464095	81.81818	C4orf10
1553320_s_at	9.58E-05	0.409786	90.90909	CDC14C
