# Supplementary figures and images for: A comparison of the molecular subtypes of triple-negative breast cancer among non-Asian and Taiwanese women
Source: Breast Cancer Res Treat. 2017 Mar 15;163(2):241–54. doi: 10.1007/s10549-017-4195-7 (PMC5410215; doi:10.1007/s10549-017-4195-7)

## Supplementary reference 3

PD1 (PDCD1)

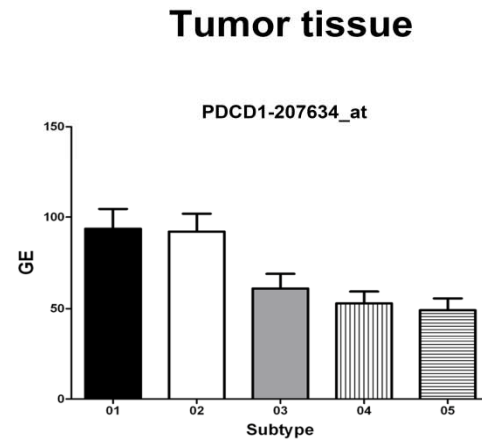

PD-L1(CD274)

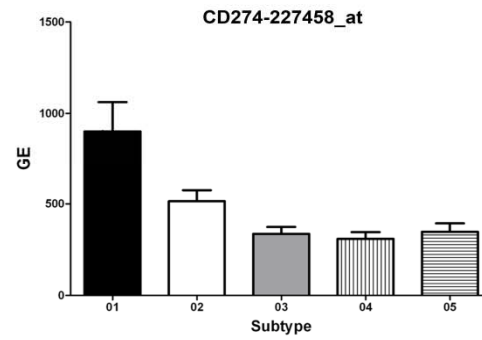

PD-L2(PDCD1LG2)

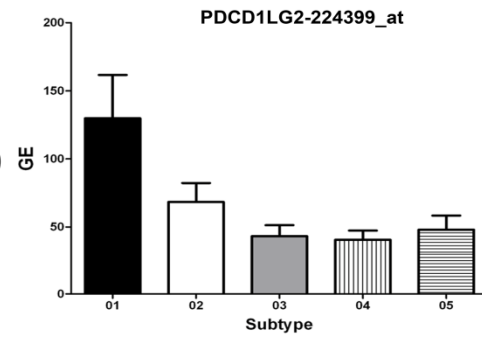

Cell lines

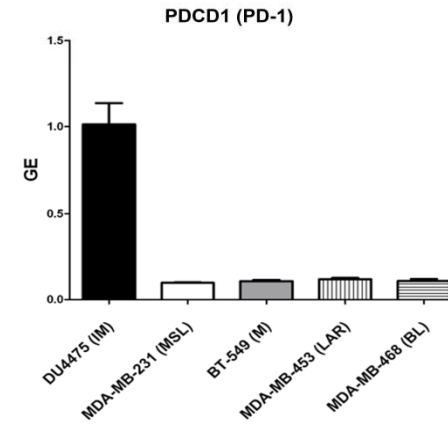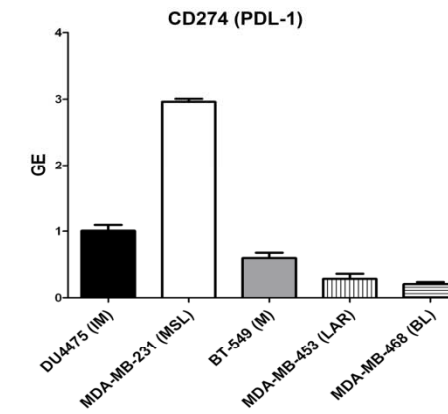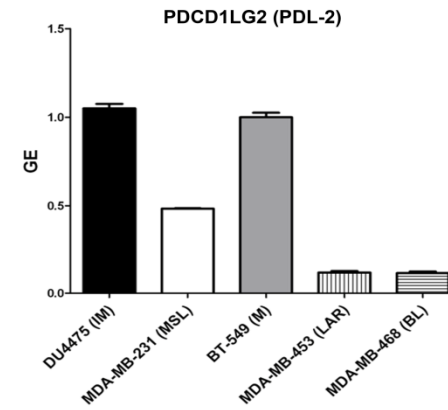

Supplement: Supplementary file 3 — Supplementary material 3 (PDF 219 kb) [file 10549_2017_4195_MOESM3_ESM.pdf]
